# Effects of Sorbitan Monostearate and Stearyl Alcohol on the Physicochemical Parameters of Sunflower-Wax-Based Oleogels

**DOI:** 10.3390/gels8080520

**Published:** 2022-08-19

**Authors:** Deepti Bharti, Doman Kim, Indranil Banerjee, Derick Rousseau, Kunal Pal

**Affiliations:** 1 Department of Biotechnology and Medical Engineering, National Institute of Technology Rourkela, Rourkela 769008, India; 2 Department of International Agricultural Technology & Institute of Green BioScience and Technology, Seoul National University, Seoul 24266, Korea; 3 Department of Bioscience & Bioengineering, IIT Jodhpur, Jodhpur 342037, India; 4 Department of Chemistry and Biology, Ryerson University, Toronto, ON M5B 2K3, Canada

**Keywords:** oleogel, gelator, wax, emulsifier, crystallization

## Abstract

A rising health concern with saturated fatty acids allowed researchers to look into the science of replacing these fats with unsaturated fatty acids. Oleogelation is a technique to structure edible oil using gelators. The present study looked for the effect of solid emulsifiers; namely, sorbitan monostearate (SP) and stearyl alcohol (SA), on the physicochemical parameters of oleogels. All the oleogels were formulated using 5% sunflower wax (SW) in sunflower oil (SO). The formulated oleogels displayed irregular-shaped wax crystals on their surface. The bright-field and polarized microscopy showed the fiber/needle network of wax crystals. Formulations consisting of 10 mg (0.05% *w/w*) of both the emulsifiers (SA10 and SP10) in 20 g of oleogels displayed the appearance of a dense wax crystal network. The SP and SA underwent co-crystallization with wax molecules, which enhanced crystal growth and increased the density and size of the wax crystals. The XRD and FTIR studies suggested the presence of a similar β’ polymorph to that of the triacylglycerols’ arrangement. The incorporation of SA and SP in wax crystal packing might have resulted in a lower crystallization rate in SA10 and SP10. Evaluation of the thermal properties of oleogels through DSC showed better gel recurrence of high melting enthalpy. These formulations also displayed a sustained release of curcumin. Despite the variations in several properties (e.g., microstructures, crystallite size, thermal properties, and nutrient release), the emulsifiers did not affect the mechanical properties of the oleogel. The meager amounts of both the emulsifiers were able to modulate the nutrient release from the oleogels without affecting their mechanical properties in comparison to the control sample.

## 1. Introduction

Fats and oils are mainly available to the body as fatty acids. They are majorly classified as monounsaturated (MUFA), polyunsaturated (PUFA), and saturated fatty acids (SFA). Most fat and fat-based products consist of a mixture of the aforementioned types of fatty acids. It is quite evident that consumption of trans and saturated fats increases the risk of cardiovascular diseases. At the same time, the use of saturated fat in food products adds to a few specific properties such as flavor, texture, and mouthfeel [1]. However, an increasing concern with the use of trans fats in food products led the U.S. Food and Drug Administration (FDA) to provide guidelines to replace saturated fats with unsaturated fats. This was done to improve the health benefits of the food products. In this regard, the food industry began its dependence on different vegetable oils for partial/complete replacement of trans fats. Long before the health concern regarding the use of saturated fats occurred, approaches such as partial hydrogenation, fractionation, and interesterification were used to form solid-like fats from vegetable oil. However, these methods lead to the formation of saturated fats during the final food production. The use of solid fat in food products provides desirable functionality, texture, and stability. Recently, scientists have explored the use of gelator molecules to form a semisolid gel using vegetable oil. Oil structuring, which is commonly referred to as oleogelation, is utilized to synthesize soft and semisolid oil-based products called oleogels [2]. With continuous growth in this field, scientists have explored different blends of vegetable oils and gelator molecules to synthesize oleogels. The formed crystal structure, morphology, and crystallinity depend on the chosen vegetable oil and gelator molecules [3]. Additionally, it has been reported that the oxidative stability of oleogels is somewhat higher than the commonly used vegetable oil [4]. Natural waxes, monoglyceride, fatty acids, and fatty alcohols are a few commonly used oleogelators [5]. Based on the food industry’s application and utilization, natural waxes often derived from plants, insects, and marine animals are suitable for forming oleogels.

Additionally, the use of plant waxes to entrap vegetable oil has now been explored widely. These gelator molecules lead to the formation of an oleogel through the direct dispersion method. The process involves melting waxes inside the vegetable oil at a temperature higher than the melting point of waxes. This step is followed by a cooling step during which a three-dimensional network formation occurs that entraps the oil. Among the food-grade waxes, sunflower wax (SW) obtained from the seeds of the *Helianthus annuus* plant has been used to structure soybean oil. SW has been reported to contain mostly wax esters (~96%), 0.2% hydrocarbons, 3.3% free fatty acids, and 0.3% long-chain alcohols, and is known to gel vegetable oil at low concentrations. Various structural characteristics of oleogels are known to be affected by the used oil type [6]. Among the frequently consumed vegetable oils, sunflower oil (SO) is a rich source of MUFAs, PUFAs, vitamin A, and carotenoids, and is widely used in the food industry due to high consumer acceptance. They contain predominantly triacylglycerols (TAGs) and a trivial amount of phospholipids.

One of the recent trends in oleogels is altering the physicochemical properties of formulated oleogels through food-grade emulsifiers [7]. Mono- and diglycerol, polysorbate, sorbitan esters, and lecithin are a few commonly used emulsifiers in the food industry [8]. Several studies have reported an ordered arrangement of stearic acid molecules after emulsifier incorporation in the oleogels [9]. Using emulsifiers inside a fat system requires extra attention, as they can alter the fat crystallization and polymorphic transitions. The fat macrostructure, including its viscoelastic properties, is often influenced by the composition of the TAGs. The amount of the TAGs, their polymorphic forms, and crystal size also can modulate the properties of the oleogels. The polymorphic transitions of TAGs occur toward the more stable polymorph from a less stable one. The emulsifier or additives often affect the process of fat polymorphism either by modifying the crystal structures during crystallization or by hindering the polymorphic transitions [10]. Sorbitan monostearate (Span 60) is a lipophilic emulsifier with a hydrophilic–lipophilic (HLB) value of 4.7 and is food-safe. It is a constituent of many available shortenings, and provides mouthfeel and softness to food products. Similarly, stearyl alcohol (SA) is a hydrophilic emulsifier with an HLB value of 15 that is generally considered safe. The HLB values of emulsifiers have shown a significant role in the stability of oil-in-water (O/W) and water-in-oil (W/O) systems [11]. However, the studies related to the role of the HLB value in oil systems are quite limited. We chose two emulsifiers, SP and SA, with different and, at the same time, extreme HLB values to study their influence on the physicochemical properties of oleogels. The effect of these emulsifiers on the SW-based oleogels has not been evaluated so far.

This work will discuss the potential role of increasing the amount of emulsifiers (SP and SA) with different HLB values on the physicochemical properties of the oleogels synthesized using 5% SW in SO. The oleogels were initially tested for their oil-binding capacity (OBC) and color properties. Metallurgical microscopy was employed to evaluate the appearance and distribution of structures on the surface. The synthesized oleogels were visualized under bright-field and polarized microscopy to analyze the microstructures. X-ray diffraction and Fourier-transform infrared spectroscopy were performed to understand the molecular interactions among the constituents of the oleogels. The formulated oleogels were further characterized via crystallization kinetics and differential scanning calorimetry studies. Further, stress relaxation was done to support the viscoelastic properties of oleogels. The oleogels were explored for their potential in nutrient release.

## 2. Results and Discussion

### 2.1. Oil-Binding Capacity

**Figure 1:**
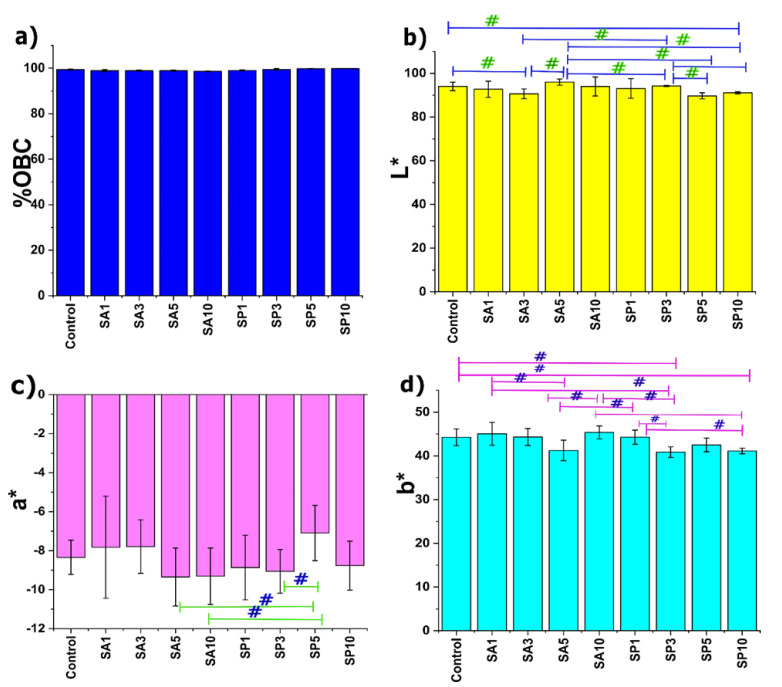
(**a**) Quantification of oil-binding capacity of oleogel. Color parameters: (**b**) L* value; (**c**) a* value; (**d**) b* value. The values in the graph are denoted as the mean of the triplicate ± standard deviation (# *p* < 0.05).

Among the different physical properties of oleogels, their oil-binding capacity (OBC) is essential. OBC helps evaluate the strength of oleogels by calculating their ability to hold the liquid oil in the three-dimensional network created by the gelator molecules. A slight and slow oil exudation is desirable in many food industries. In this study, all oleogels showed an OBC greater than 99%. The critical gelation concentration (CGC) of 5% of wax was thus sufficient for gelling the SO. This perfectly correlated with previous studies in which sunflower wax-based oleogels showed OBC values higher than 99% [12]. It was also interesting to note (Figure 1) that there was no potential influence of the used amount of emulsifiers upon the OBC. Crystal morphologies such as needle-like or fibrous structures are known for effective oil structuring [13]. The emulsifiers might not have caused any significant alteration in the morphology of the crystals, and therefore, no influence was observed on the OBC.

### 2.2. Colorimetry Study

Color is a crucial parameter of food products that often influences consumer selection. The colorimetric analysis calculates color values similar to those perceived by the human visual system. The results concerning the color analysis of oleogels are represented in Figure 1. The majority of the color measurement systems are based upon the CIE system, which categorizes color based on luminance and two color parameters. Although the emulsifiers did not significantly affect the oil-binding capacity, they affected a few of the color parameters. The color parameters used in the study included the L* value, which is the measure of brightness/lightness and ranges from 0 (black) to 100 (white). The L* value of the control (94.04 ± 1.93) was an indication of a potentially bright and luminous formulation. Inclusion of SA in the case of SA1 showed a similar lightness to the control, although SA3 showed a significant reduction in the L* value from that of the control. The possible reason behind this could be the presence of larger crystals or crystal aggregation (marked in red) on the surface of the SA3 oleogel, which will be confirmed through the surface topology in the next section. A further increase in the amount of SA in the case of SA5 showed a similar L* value to the control. Nevertheless, this increase was significant compared to SA3. There is a possibility that adding 5 mg of SA to the formulated oleogel resulted in a light-colored formulation. Among the Span 60 formulations, SP1, SP3, and SP5 appeared similar to the control in their L* values. SP10 showed a marked reduction from the control. SP1 and SP3 seemed to be similar among the samples. The decrease in the luminous value of SP5 and SP10 from SP3 was statistically significant. A higher L* value; i.e., above 90, indicated that the formulations consisted of smaller wax crystals that reflected more light due to a larger surface area [14]. The two-color descriptors used in this study were the values of a* (green vs. red) and b* (blue vs. yellow), which range from −120 to +120. All the oleogels prepared in the study displayed a negative a* value corresponding to green tones. The emulsifier-added oleogels displayed a similar a* value to that of the control. Further, observing positive b* values among all the formulations indicated the presence of yellowness in the samples. The SA-added oleogels displayed no significant differences in their b* values compared to the control. In addition, few variations were observed among the samples, except for the decrease in the b* value of SA5 from SA1 (*p* < 0.05). SP1 showed a similar b* value to the control in the SP-added formulations. A further rise in the emulsifier content showed a substantial decrease in yellowness in SP3 from the control and SP1. SP5 displayed a similar b* value to the control, SP1, and SP3. At the highest content of this emulsifier; i.e., SP10, there was a significant reduction in the b* value from the control and SP1.

### 2.3. Microscopy

#### 2.3.1. Surface Topology

**Figure 2:**
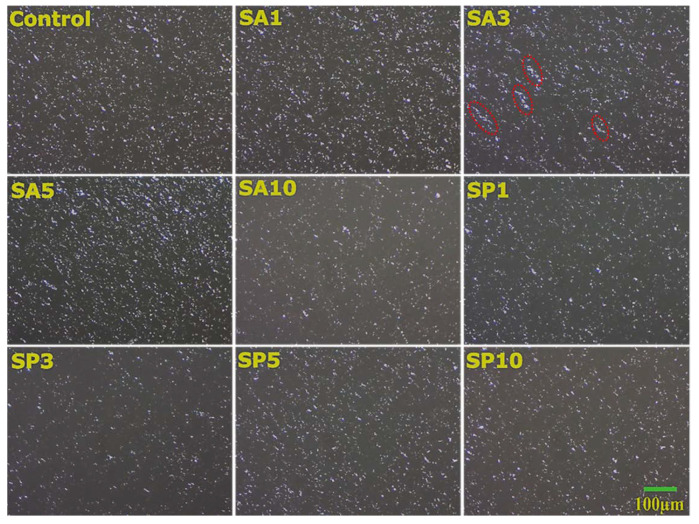
The appearance of bright platelet crystals on the surface of oleogels.

The surface topology of the oleogels displayed glowing crystals distributed throughout the surface. The structuring of SO using SW resulted in platelet crystals (Figure 2) that differed in size and density after addition of both emulsifier types. The surface images also indicated the semicrystalline nature of oleogels composed of crystalline and amorphous zones. The homogeneous distribution of a platelet structure was considered to be a desirable characteristic. At the lowest amount of SA in oleogels, the wax crystals appeared similar to the control in their distribution and size. However, the crystals grew in density and size at an increased amount of SA through SA5. The conjoint crystal on the surface of SA3 are marked in red in Figure 2. On the contrary, SA10 showed minimal crystals on its surface. SA5 showed better crystal distribution on its surface among the SA-included samples. The inclusion of SP in the case of SP1 showed a similar topology to the control; however, SP3 showed a marked reduction in the crystal density. The surface topology appeared identical to control at the higher SP amount; i.e., SP5 and SP10.

The crystal topology was also visualized under a metallurgical microscope (Figure S2). The distribution of crystalline and amorphous regions was similar to that found in the previous study. Interestingly, the morphology of the wax crystals was different. At a higher magnification, the surface topology was filled with irregularly shaped fat crystals that appeared globular in stereomicrographs.

#### 2.3.2. Microstructure Analysis

The microstructures of the formulated oleogels were visualized under an optical microscope. The optical microscope’s bright-field and polarized modes could portray the crystal network that resulted in the vital oil-binding capacity of these oleogels. All the formulations showed an overall appearance of fiber-like crystals, which varied in number and shape based on the amount and type of emulsifier used (Figure S3). The appearance of fiber-shaped crystals could be due to long-chain wax esters [15]. Earlier studies displayed that fiber- or needle-like structures formed from beeswax and rice bran wax efficiently entrapped vegetable oil at 5 wt %. Low-molecular-weight organic gelators such as waxes are capable of forming self-assembled networks. The microstructures formed by waxes are also termed self-assembled fibrillary networks (SAFiNs) [16]. It was rational to assume that the wax crystal in the oleogels formed a solid network analogous to the fat crystal network. On close observation, it was seen that the control sample consisted of a dense fiber network with small fiber lengths. The gelation of the oil phase was due to the high aspect ratio (length:diameter) of the fiber morphology of the crystals [17]. The high surface area of the fiber morphology is crucial for pronounced contact between the crystals, and can be associated with the solid and elastic properties of gels [17].

The addition of SA caused a reduction in the density of the self-assembled fibers in SA1 and SA3. However, the inclusion of SA resulted in increased fiber length in all the cases. There was a possibility of an increase in the amount of crystalline surface with the increased inclusion of SA, which aided in crystal growth. Interestingly, in SA5, there were distinct fibers with an increased length. SA10 showed long fibrous structures with a comparatively greater thickness among all the SA-included samples. On addition of SP into the oleogels, SP1 and SP3 showed the presence of longer fibers than the control. The arrangement of wax fibers in SP5 and SP10 could not be clearly visualized through the bright-field illumination.

**Figure 3:**
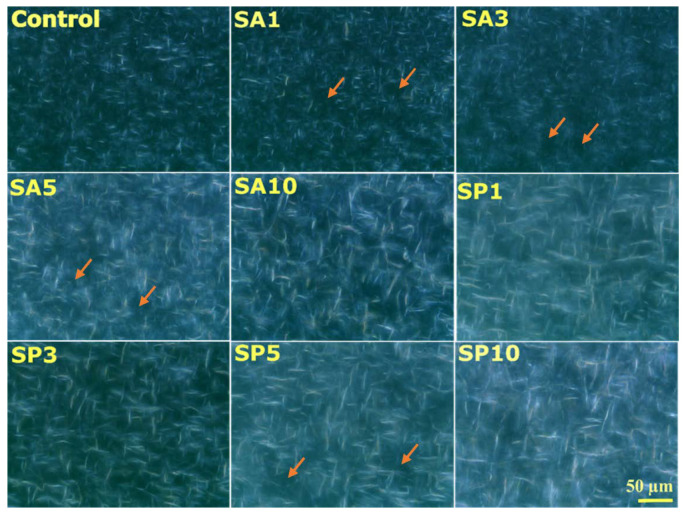
Three-dimensional network of needle-like wax crystals in oleogels.

The shape and structural arrangement of these crystals could be better obtained in a non-quantitative manner through polarized-light microscopy (Figure 3). The crystals were birefringent and appeared white, contrary to the liquid oil, which was black. The variability among the properties of oleogels could be due to the crystal size, crystal habit, and crystal–crystal interactions. Crystallite size is often linked to important functional properties such as the texture of the oleogels [18]. The presence of fibrous or needle-like arrangements of wax crystals in the oleogel system was confirmed through the polarized micrographs. This needle-like arrangement further indicated the possibility of the presence of β’ crystals similar to fats [19]. These micrographs displayed a fat network arrangement. The high surface area of this needle/fiber-type morphology opened the possibility for vast interaction between the observed microstructures and oil [17]. Formulations such as the control, SA1, and SA3 showed the presence of a few bright crystals and very few amorphous regions (marked in orange). There was an evident rise in the amorphous regions of SA5. Interestingly, SA10 showed many long bright wax crystals that gave a rigid crystal-packing appearance. The inclusion of SP in the oleogels showed very few bright crystals, which were comparatively smaller than those in the control. Similar to SA5, SP5 also displayed significant zones that were regarded as amorphous regions. At the highest amount of SP; i.e., SP10, the wax crystals appeared the brightest among the formulations. The possible explanation for the better crystal appearance and arrangement at the higher emulsifier content was their ability to undergo co-crystallization along with the wax molecules, which is crucial in many food applications [20].

### 2.4. Molecular Characterizations

#### 2.4.1. XRD Analysis

XRD is widely used to study the phenomenon of polymorphism in the fat system. The XRD diffractogram and percentage crystallinity of the formulated oleogels is shown in Figure S4. The diffractograms represented broadband, which peaked at ~22° 2θ. A second sharp peak was observed at ~25° 2θ, and a third smaller sharp peak at ~27° 2θ. The broad peak was due to the amorphous regions, while the sharp peaks were due to the crystalline regions. The increased intensity after the emulsifier incorporation in most formulations suggested their positive contribution to lateral packing [21].

The diffraction profiles were loaded into Origin Pro software for deconvolution using the Gauss peak fitting function for obtaining accurate peak positioning and calculation of other dependent parameters. These parameters included crystallite size, lattice strain, and dislocation density (Table 1). The diffractogram of the control displayed a broad peak at 22.82° 2Ө, 25.06° 2θ and 27.85° 2θ. The d-spacing values of these parameters were calculated as 4.52 Å, 4.12 Å, and 3.71 Å, respectively. Two of these d-spacing values (4.12 Å and 3.71 Å) were previously reported for the presence of β’ polymorphs of triacylglycerols, and thus displayed similarity with the wax crystal. The β’ polymorph displayed orthorhombic subcell packing, which was denser and seamless [22]. The inclusion of emulsifiers showed a slight shift in the positions of the mentioned peaks. The observed shifting may have occurred due to the incorporation of emulsifier molecules in the subcell lattice. The inclusion of SA showed a decreased crystallite size from control through SA3, although formulations SA5 and SA10 showed a larger crystallite size. SA5 and SA10 also displayed a lower lattice strain, which affirmed minor crystal defects in the system. A similar observation was evident in the PLM images of SA10. Surprisingly, inclusion of SP at lower amounts; i.e., in SP1 and SP3, displayed a drastic reduction in the crystallite size, which began rising upon a further increase in the amount of the emulsifier. The possible explanation for such a drastic reduction could be an occurrence of rapid crystallization inside the mentioned formulations. SP10 showed a lower lattice strain and dislocation density among the SP incorporated samples, favoring stable lateral packing. Additionally, SA10 showed the largest crystallite size, and SP10 displayed the highest crystallinity among all the formulations. The occurrence of co-crystallization of emulsifiers and the wax esters may have led to crystal growth, and thus better crystallinity.

#### 2.4.2. FTIR Analysis

The FTIR spectra of the oleogels were investigated to examine different interactions between the constituents of the oleogels and used emulsifiers. The spectra of the raw materials (SO, SW, SP, and SA) are also presented in the Supplementary Materials (Figure S5). SP is an ester made from sorbitan and stearic acid. The sorbitan emulsifier (SP) comprised polar groups in their hydrophilic portion; however, the hydrophobic moieties included esters, fatty alcohols, and fatty acids. A peak at 3300 cm^−1^ in the spectra of SP was attributed to the O-H stretching. The appearance of sharp peaks at 2916 cm^−1^ and 2848 cm^−1^ resulted from the aliphatic C-H stretching. The hydrophobic moieties of SP also comprised C = O and C-O from ester groups, which were confirmed from the spectral bands positioned at 1734 cm^−1^ and 1218 cm^−1^ [23]. A spectral peak at 1467 cm^−1^ represented the C-H bending vibration. In the spectra of SA, the aliphatic hydroxyl (O-H) group was confirmed through the peaks at 3328 cm^−1^ and 1061 cm^−1^. The sharp bands at 2916 cm^−1^ and 2846 cm^−1^ were attributed to the asymmetric stretching vibration of alkyl C-H, and the peak at 1463 cm^−1^ resulted from the C-H bending. The obtained spectra of SO and SW followed a previously reported work [24].

FTIR spectroscopy was further explored to confirm the interactions among the various components of the oleogels (Figure S6). The IR spectra of both the SA- and SP-treated oleogels appeared similar to that of the control. Unlike SP and SA, there was no O-H stretching peak in the oleogels. A medium spectral peak at 3005 cm^−1^ resulted from the C-H stretching vibration of alkene. The appearance of this peak was due to the unsaturation from the linoleic and oleic acid present in SO [25]. Spectral signals at 2920 cm^−1^ and 2854 cm^−1^ may have been related to the C-H stretching vibration in alkanes. These peaks were contributed by vegetable oil. The medium spectral peaks at 1461 cm^−1^ and 724 cm^−1^ were due to alkane’s angular bending and rocking vibrations. One interesting finding for these two peaks was their ability to predict the packaging inside the lipid system. The spectral peaks at 1461 cm^−1^ and 724 cm^−1^ further confirmed the orthorhombic subcell packing, which was first confirmed through the XRD studies described above [26]. The prominent spectral peak at 1744 cm^−1^ in the oleogels arose from the abundant carbonyl functional group of ester present in SO and SW [27]. The occurrence of peaks in the range of 1210 cm^−1^−1163 cm^−1^ was assigned to the C-O stretching vibration of the ester. At the chosen spectral resolution of 4 cm^−1^, there was no observed shift among the spectra of all the formulations. This observation further confirmed the absence of any difference in the chemical interactions among the emulsifier-added oleogels compared to the control oleogel. A possible explanation for this is the lower amount of emulsifiers used to prepare the formulations.

### 2.5. Thermal Analysis

#### 2.5.1. Crystallization Kinetics

The formation of crystals from the molten state is a first-order transition and is defined as crystallization. The three phases of lipid crystallization include nucleation, crystal growth, and maturation. The crystallization is mainly based on the nucleation process, which is the initial stage in forming crystals. A rapid rate of crystallization results in the formation of a diffuse crystalline phase associated with low-energy polymorphs. However, the growing crystals are arranged into a consistent three-dimensional lattice under a slow crystallization rate [28]. A temperature vs. time plot is considered suitable for understanding crystallization kinetics (Figure S7). The plot can be segregated into the initial, intermediate, and final stages of saturation. There is a sharp decline in the graph from temperature after the marked pink arrow. This marks the nucleation and beginning of the secondary crystallization. In the control sample, the initiation of secondary crystallization was marked around 1000 s. Including an emulsifier in the system may have added imperfections to the crystal lattice, thus disturbing this onset. The emulsifiers’ amount, composition, and mechanism of action could affect the nucleation or/and growth. The addition of SA shortened the onset time in all the formulations compared to the control. The use of SA may have accelerated the early crystallization phase through heterogeneous nucleation with wax molecules [29]. This would eventually affect the crystallization kinetics and polymorphic transition in the formulated oleogels [30]. The inclusion of SP resulted in a rapid onset of secondary crystallization in SP1 and SP10. The blue arrow in the figure marks the accomplishment of the isothermal stage in the crystallization kinetic curve. It was interesting to observe that although the emulsifier-incorporated oleogels showed significant differences in the onset of secondary crystallization (Table 2) compared to the control, there were not many variations in the attainment of the isothermal stage.

The crystallization curve was further modeled using an exponential decay function (Equation (1)) to calculate the rate of crystallization (k). There was an overall increase in the crystallization rate of all the formulations compared to the control (Table 2). Among the SA formulations, the crystallization rate increased through SA3 and decreased afterward. The lowest crystallization rate (for SA10) might have resulted in forming a stable crystal arrangement and crystallite sizes. This was also evident in the PLM micrographs and XRD data. Among the SP formulations, SP1 showed the highest rate of crystallization, which explained the reduction in their crystallite size as shown in the XRD data. The rest of the SP formulations displayed a lower crystallization rate, and there was not much variation observed in the rate. Interestingly, a lower crystallization rate in SP10 also correlated with brighter crystals and a relatively larger crystallite size. The more stable phases usually were slow to crystallize.

(1) $$y=ae^{-kt}$$

where *t* is the time (s), a is the initial temperature (50 °C), and *k* is the rate of crystallization (°C/msec).

#### 2.5.2. Differential Scanning Calorimetry

The cooling and heating thermograms of the emulsifier-incorporated oleogels are shown in Figure S8. The modification of the physical state and polymorphic transformation of the oleogels can be visualized through the endothermic and exothermic curves of the DSC. The SW used in the study had a high melting point (~70 °C) [31]. Most natural waxes, including SW, displayed different crystallization and melting patterns inside the vegetable oil. The crystallization and melting temperatures of wax-based oleogels were lower than those of neat waxes. The melting (green) and crystallization (pink) thermograms of oleogels were visualized. The onset of melting (T_0m_) and crystallization (T_0c_) for all the formulations was seen between 46−50 °C and 60−63 °C, respectively. The curve was further evaluated to calculate the events of onset temperature, peak temperature, and area under the peaks for the melting and crystallization cycles (Table 3). It was seen that the melting peak (T_m_) of the control was marked around 62 °C. Oleogels formulated using SW and olive oil were previously reported to have a T*_m_* of 58 °C. The inclusion of both the emulsifiers showed slight shifts in the Tm and enthalpy values (∆H*_m_*). The observed melting point range was attributed to the melting of TAGs. The values of Tm and ∆H_m_ helped in evaluating the thermal strength and stability of the oleogels [32]. Higher ∆H_m_ values for SA3, SA10, and SP10 correlated with their better gel strength. A larger crystallite size and better thermal stability of SA10 and SP10 also highlighted the possibility of a melt-mediated transition from β’ to β polymorphs similar to TAGs, associated with the formation of large crystals [33]. The peak temperature of crystallization (T_c_) was found at ~60 °C for all the formulations. A constant gelator concentration for all the formulations may have resulted in a similar T_c_ [34]. In Table 3, it is quite evident that the ∆H*_m_* for every sample was lower than its respective crystallization enthalpy (∆H_c_). This observation was in accordance with a previously reported work [31]. This phenomenon (∆H_c_ > ∆H_m_) is often termed hysteresis and is related to the enthalpy change due to the dissolution of crystals during melting. The appearance of a single intense peak on both curves was suggestive of the dominance of wax esters in the crystallization of oleogels [35].

### 2.6. Mechanical Studies

The viscoelastic properties of the oleogels were evaluated through stress-relaxation curves (Figure S9). The maximum attained force (F_0_) in the SR profile helped us to understand the firmness of the formulations. As a constant strain was applied during the relaxation period, the force value declined exponentially until it became constant. Toward the end of the relaxation process, the force value was represented as F_R_, which provided an idea about the residual elastic force in the formulation. Further, F_0_ and F_R_ were considered in the calculation of % SR (Equation (5)), which depicted the ability of the system to absorb the energy when placed in strained conditions. The F_0_, F_R_, and % SR values are presented in the Supplementary Materials (Table S1). The formulations displayed no significant differences in their firmness, residual force, or % SR. Interestingly, though the formulated oleogels had different microstructural arrangements, crystallite sizes, and crystallization kinetics, these properties did not influence their mechanical strengths. The mechanical properties could also be affected by the lattice strain, which remained nearly similar in this study [36]. This similarity in the strain value also suggested that the formed crystals must have accommodated themselves in a similar manner, and therefore the mechanical properties remained preserved.

### 2.7. Nutrient Release

**Figure 4:**
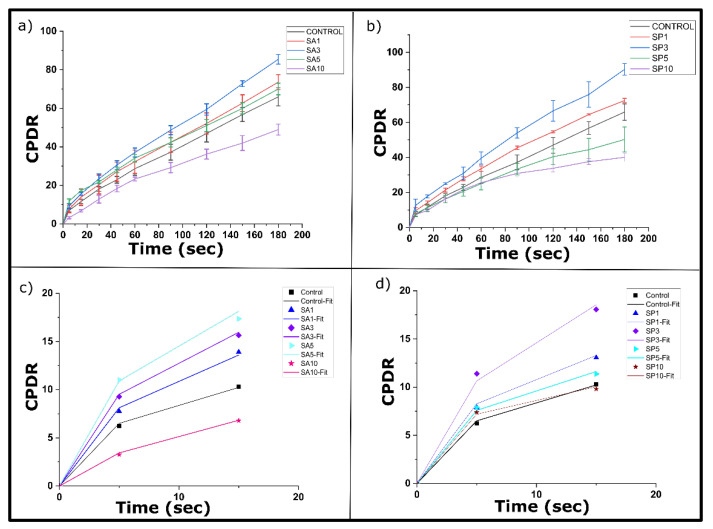
Nutrient release profiles of (**a**) SA oleogels and (**b**) SP oleogels. PS model fitting of (**c**) SA oleogels and (**d**) SP oleogels.

Curcumin, a biomolecule with functional moieties, is a potent nutraceutical agent [37] and a natural dietary component. Curcumin has low aqueous solubility and low bioaccessibility in the gastrointestinal (GI) system. The possible reason behind this is its release from the carrier matrix and simultaneous absorption in the GI tract. Oleogels, as a hydrophobic matrix, can dissolve hydrophobic biomolecules and further provide protection and sustained release [38]. The release profiles of curcumin from the prepared oleogels are displayed in the Figure 4. It was found that the formulations SA3 and SP3 showed a higher (*p* ≤ 0.05) CPDR for 180 min compared to the control. On the other hand, SA10 and SP10 showed a lower CPDR than the control during a similar period. This indicated the possibility of sustained release of curcumin from the SA10 and SP10 oleogels. The presence of a larger crystallite size in these formulations, which was confirmed through XRD studies (Table 1), could have been the reason for this low release. The curcumin molecules might have been entrapped within these larger crystallites during the crystallization process [39]. The sustained release in SA10 and SP10 would then result in an effective and controlled release of curcumin. This sustained release could further reduce the frequency of dosing [40].

The diffusion curve was fitted to Peppas–Sahlin (PS) model (Equation (2)) for the initial data points. The PS model considered that the drug release from the polymer could either be due to the Fickian diffusion or diffusion mediated by polymer chain relaxation [41]. This model helped to understand the contribution of the aforesaid diffusion mechanisms during the drug-release process through different parameters (Table 4) [42]. Among the SA oleogels, the Fickian diffusion constant values (K_d_) were increased for SA3 and SA5 compared to the control. The possible explanation behind this rise could be the presence of amorphous regions, as seen in the PLM micrographs. On the contrary, the K_d_ values were reduced significantly in SA10 from the control, supporting the sustained Fickian release. The values of K_r_ were reduced (*p* < 0.05) in SA10 and SP10 from the control, suggesting a negligible role of matrix relaxation for the curcumin release in these cases [43]. In all the samples, the K_d_ values were higher than the K_r_ values, which supported the significant contribution of Fickian diffusion to relaxation-mediated diffusion toward the observed nutrient release from the oleogels.

(2) $$F=K_{d}t^{m}+{\;K}_{r}t^{2m}$$

where K_d_ and K_r_ are diffusion and kinetic relaxation constants, respectively, and *m* is the slope that represents the diffusion coefficient.

## 3. Conclusions

The present work aimed to formulate an oleogel through the direct dispersion of SW in SO. The potential roles of food-permissible emulsifiers with different HLB values have long been explored in fats; however, in a wax-based oleogel system, such studies are limited. Therefore, the formulation was further modified through the inclusion of solid hydrophobic (SP) and hydrophilic (SA) emulsifiers. Synthesized oleogels appeared white and smooth to the touch. Various physicochemical properties of the oleogels were evaluated through color analysis, visualization of surface topology, microstructural arrangement, molecular interaction, thermal properties, and mechanical parameters. The surface topology of the oleogels revealed the presence of globular fat structures, which appeared irregular in shape when magnified. Under the bright-field and polarized-light microscopy, the oleogels displayed fibrous/needle-like arrangements. Formulations with the highest amount (10 mg) of both the emulsifiers; i.e., SA10 and SP10, had bright fat crystals with dense packing, which assisted in gel stability. The results of the XRD study further confirmed the previous observation of the micrographs. SA10 showed the largest crystallite size, and SP10 displayed the highest crystallinity among all the formulations. All the formulations displayed similar FTIR spectra, further suggesting an insignificant effect of the emulsifiers on the molecular interactions. The occurrence of temperature-dependent polymorphs allowed the exploration of the thermal properties of the formulations. A lower crystallization rate in SA10 and SP10 indicated stable and accurate packing. The higher ∆H_m_ values obtained from the DSC curve for SA10 and SP10 correlated with their better gel strengths and thermal properties. Additionally, these formulations displayed a sustained release of curcumin for a duration of 180 min. An interesting observation was made when the oleogels were evaluated for their mechanical properties. Although the formulations displayed variations in their surface topologies, wax fiber/needle arrangements, crystallite sizes, thermal parameters, and nutrient release profiles, these variations did not affect the firmness, residual elastic forces, or stress-relaxing potentials. The observed changes in microstructures and crystallite size could be due to the co-crystallization of the used emulsifier with the wax molecules. With the growing demand for unsaturated fats, SW-based oleogels can potentially act as a solid fat replacer. Additionally, it is possible to tailor the physicochemical properties of these edible oleogels. In short, it can be concluded that although the emulsifiers were used in meager amounts in these oleogels, we were able to modulate the nutrient release from the oleogels without affecting their mechanical properties in comparison to the control sample.

## 4. Materials and Methods

### 4.1. Materials

The refined sunflower oil (Fortune Sunlite, Kutch, India) is commercially available and was purchased from a local supermarket to synthesize the oleogels. Sunflower wax (melting point ~75 °C) was purchased from Vijay Impex, India. Pure stearyl alcohol and sorbitan monostearate (Span 60) with melting points of ~60 °C were procured from Loba Chemie Pvt. Ltd., Mumbai, India.

### 4.2. Oleogel Synthesis

Oleogel of SO containing 5% (*w/w*) of SW was synthesized as per a previously mentioned protocol [14]. To accurately add small amounts of the emulsifiers (SA and SP) to the oleogels, a 0.1% stock solution of both the emulsifiers in SO was prepared separately. The SA-incorporated oloegels were labeled as SA1, SA3, SA5, and SA10 for 1 mg (0.005% *w/w*), 3 mg (0.015% *w/w*), 5 mg (0.025% w/w), and 10 mg(0.05% *w/w*) of the emulsifiers, respectively. Similarly, SP-incorporated oleogels were labeled as SP1, SP3, SP5, and SP10. The emulsifier-added oleogels were prepared by adding the required amount of the emulsifier stock solution so that the final concentration of SW was 5% (*w/w*) within the oleogels.

### 4.3. Quantification of OBC

The integrity of each formulated oleogel was evaluated by measuring the OBC, which is considered an important property of oleogels. The quantification was done by centrifuging the oleogels to separate unbound oil, if any. A 2 mL centrifuge tube was accurately weighed (w) and filled with 1 mL of molten oleogel. Then, the centrifuge tube was stored at 4 °C for 24 h. The oleogel-filled tube was weighed (w’) and centrifuged at 10,000 rpm for 15 min. The released oil was removed using tissue paper. Thereafter, the tubes were weighed again (w”). The c and % OBC were calculated using the formulas below [44]:

(3) $$\%\;\mathrm{Oil}\;\mathrm{loss}=\{\frac{\left[\left(w^{\prime }-w\right)-\left(w^{\prime \prime }-w\right)\right]}{\left(w^{\prime }-w\right)}]*100$$

(4) % OBC=100−% Oil released

### 4.4. Colorimetric Study

The role of the colorimetric analysis was to characterize the formulated oleogels based on their colors. This study was performed in an in-house-built colorimeter using a white light source (warm white light-emitting diode) [45]. The color parameters were calculated in the CIE-L*a*b* color space. The brightness or opacity of the oleogels was based on the L* values. Additionally, a* and b* were chromatic components.

### 4.5. Microscopic Studies

#### 4.5.1. Surface Features

The surface topographies of the oleogels were obtained using a stereo zoom microscope (model: SM-2TZ; make: Amscope, Irvine, CA, USA) and metallurgical microscope (AmScope, ME580TA, Irvine, CA, USA). For this purpose, ~5 g of each molten oleogel sample was placed in a 35 mm petri dish, where it was allowed to solidify as per the protocol described in Section 2.2. The solidified oleogels were then used for the surface analysis.

#### 4.5.2. Microstructural Observation

The microstructural features of the oleogels were analyzed through an upright bright-field compound microscope (DM750, Leica Microsystems, GmbH, Wetzlar, Germany). The device was also externally connected to an in-house-built polarizer. For the analysis, a drop of molten oleogel was placed on a glass slide and subsequently covered using a coverslip. The microstructure morphology and the arrangement of the wax crystals inside the oleogel system were assessed after 2 h of incubation at 25 °C in both bright-field and polarizing modes.

### 4.6. Molecular Studies

#### 4.6.1. Fourier-Transform Infrared (FTIR) Spectroscopy

An FTIR spectrophotometer (Alpha-E; Bruker, Billerica, MA, USA) was used to obtain the IR spectra in attenuated total reflectance (ATR) mode with a spectral resolution of 4 cm^−1^. The scanning was done in the wavenumber range of 600–4000 cm^−1^.

#### 4.6.2. X-ray Diffraction Analysis (XRD)

The XRD patterns of the oleogels were obtained by using an X-ray diffractometer (model: Bruker D8 Advance, Austin, TX, USA). The instrument consisted of a Co-Kα radiation source (wavelength = 1.79 Å). The X-ray tube was operated at a voltage of 35 kV and a current of 25 mA during the analysis. The scans were obtained at a scan rate of 5° 2θ/min in the 2θ range from 5° to 50°. Different parameters such as d-spacing (d), the crystallite size (D), lattice strain (ϵ), and dislocation density (δ) were calculated through the diffractogram [24].

### 4.7. Thermal Analysis

#### 4.7.1. Crystallization Studies

A total of 10 grams of molten oleogels was placed in glass bottles and heated at 80 °C. The crystallization kinetics study was performed as reported earlier [46]. Temperature sensors were placed inside the oleogel-filled glass bottles in a refrigerated water bath (5 °C). The changes in the temperature profile were recorded for 90 min.

#### 4.7.2. Differential Scanning Calorimeter (DSC)

A total of 10 mg of each oleogel was sealed in an aluminum pan using a pierced lid. The DSC (200 F3 DSC, Maia, Netzsch, Burlington, VT, USA) was operated at a thermal scan rate of 5 °C/min to obtain the melting and crystallization curves of the oleogels. A thermal program was set for each oleogel, including a melting profile from 0 °C to 100 °C. Similarly, a cooling profile was captured in the range of 100 °C to 0 °C. In between the melting and cooling stages, the oleogels were maintained at 100 °C for 5 min.

### 4.8. Mechanical Characterization

An HDplus texture analyzer (Stable Microsystems, Surrey, UK) was employed to examine the stress-relaxation (SR) profiles of the oleogels. The oleogels were formulated in propylene beakers for the SR study. With an initial trigger force of 5 g, oleogels were penetrated at a rate of 0.5 mm/s and a constant strain of 1 mm. The penetration was achieved with an acrylic male conical probe (45° angle). Thereafter, the strain was maintained for 60 s. Changes in the force values were recorded, after which the probe was taken back to its usual height. The % SR was calculated for each sample using the formula below:

(5) $$\%\mathrm{SR}=\frac{F_{m}-F_{r}}{F_{m}}\times 100$$

where F_m_ is the maximum achieved force in SR and F_r_ is the residual force

### 4.9. Nutrient Release

The dissolution apparatus (model: DA8000, Labindia Instruments Pvt. Ltd., Mumbai, India) was used to study the in vitro curcumin (a model nutrient) release. The study was conducted as per the *United States Pharmacopoeia* (type I; basket-type) method. Curcumin-loaded oleogels were prepared through the trituration method. Each oleogel consisted of 5 mg/g (*w/w*) of curcumin. A total of 1.0 g of the curcumin-loaded oleogels were loaded into the dissolution basket. Double-distilled water (500 mL) maintained at 37 ± 0.5 °C was used as the dissolution media. The basket was rotated at 50 rpm, and 5 mL of the dissolution media was withdrawn at regular intervals and replaced with fresh water. The analysis of the curcumin content was conducted using a UV–vis spectrophotometer (model: Shimadzu 1800, Japan). The time-dependent curcumin release profile was plotted as cumulative percent drug release (CPDR).

### 4.10. Statistical Analysis

The analysis of the various experimental data was conducted in triplicate and reported as the mean ± standard deviation. The analysis was performed using a Student’s *t*-test in IBM SPSS Statistics (version 20, IBM, Armonk, NY, USA).

## Figures and Tables

**Table 1:** Parameters obtained after deconvolution of XRD peaks.

Formulations	Peak	Peak Position (° 2θ)	FWHM (° 2θ)	d-Spacing (Å)	Crystallite Size (nm)	Lattice Strain	Dislocation Density (δ) × 10 ^17^ lines/m^2^
Control	1	22.82	8.102	4.52	1.21	0.17	0.68
	2	22.82	4.09	4.52	2.40	0.08	0.17
	3	25.06	0.42	4.12	23.35	0.00	0.00
	4	25.06	14.17	4.12	0.70	0.27	2.04
	5	27.85	0.43	3.71	23.18	0.00	0.00
	Average	-	-	**4.20**	**10.16**	**0.11**	**0.58**
SA1	1	21.19	10.07	4.86	0.97	0.23	1.06
	2	22.23	4.61	4.63	2.13	0.10	0.22
	3	24.69	0.53	4.18	18.36	0.01	0.00
	4	24.69	8.88	4.11	1.11	0.17	0.81
	5	27.47	0.53	3.76	18.53	0.01	0.00
	Average	-	-	**4.32**	**8.22**	**0.11**	**0.42**
SA3	1	23.09	8.46	4.46	1.16	0.18	0.74
	2	23.09	4.29	4.46	2.29	0.09	0.19
	3	25.33	0.42	4.07	23.39	0.00	0.00
	4	25.33	14.42	4.07	0.69	0.27	2.10
	5	28.10	0.44	3.68	22.41	0.00	0.00
	Average	-	-	**4.15**	**9.98**	**0.11**	**0.60**
SA5	1	23.01	9.02	4.48	1.09	0.19	0.84
	2	23.01	4.47	4.48	2.20	0.09	0.20
	3	25.13	0.38	4.11	25.33	0.01	0.00
	4	27.84	0.45	3.71	21.99	0.01	0.00
	5	27.84	11.96	3.71	0.83	0.21	1.45
Average	Average	-	-	**4.10**	**10.28**	**0.10**	**0.50**
SA10	1	22.31	9.13	4.62	1.08	0.20	0.86
	2	22.85	4.58	4.51	2.15	0.09	0.21
	3	25.14	0.40	4.10	24.58	0.00	0.00
	4	25.14	10.73	4.10	0.92	0.21	1.18
	5	27.87	0.43	3.71	22.96	0.00	0.00
	Average	-	-	**4.21**	**10.33**	**0.10**	**0.45**
SP1	1	22.62	10.02	4.56	0.98	0.22	1.04
	2	22.86	4.78	4.51	2.05	0.10	0.23
	3	25.15	0.39	4.10	4.10	0.10	0.05
	4	27.66	0.96	3.74	10.25	0.01	0.00
	5	27.66	6.52	3.74	1.52	0.11	0.43
	Average	-	-	**4.133**	**3.78**	**0.11**	**0.35**
SP3	1	22.93	9.73	4.49	1.01	0.20	0.98
	2	22.93	4.55	4.49	2.16	0.9	0.21
	3	25.18	0.41	4.10	2.16	0.09	0.21
	4	27.95	0.47	3.70	20.98	0.00	0.00
	5	27.95	11.30	3.70	0.88	0.19	1.29
	Average	-	-	**4.10**	**5.44**	**0.12**	**0.54**
SP5	1	23.20	7.93	4.45	1.24	0.16	0.65
	2	23.20	4.39	4.45	2.24	0.09	0.19
	3	25.36	0.43	4.07	22.5	0.00	0.00
	4	25.36	13.58	4.07	0.73	0.26	1.87
	5	28.12	28.12	3.68	21.37	0.00	0.00
	Average	-	-	**4.14**	**9.616**	**0.11**	**0.54**
SP10	1	21.88	9.66	4.71	1.02	0.21	0.96
	2	22.75	4.63	4.53	2.12	0.10	0.22
	3	25.10	0.41	4.11	23.71	0.00	0.00
	4	25.10	9.13	4.11	1.08	0.17	0.85
	5	27.85	0.45	3.71	21.97	0.00	0.00
	Average	--	--	**4.24**	**9.98**	**0.10**	**0.10**

**Table 2:** Parameters obtained through the crystallization kinetics of oleogels.

Formulations	Temperature vs. Time		Exponential Decay Model
	Onset of Secondary Crystallization (s)	Time to Reach Thermal Equilibrium (s)	Rate of Crystallization (°C/ms)
Control	999	2217	0.99
SA1	765	2089	1.50
SA3	633	2109	1.60
SA5	783	2169	1.32
SA10	688	2393	1.26
SP1	530	2199	1.76
SP3	991	2568	1.11
SP5	910	2515	1.14
SP10	720	2288	1.17

**Table 3:** Melting and crystallization parameters obtained from the DSC thermograms.

Formulations	Melting			Crystallization			Degree of Supercooling (°C)
	T_0m_ (°C)	T_m_ (°C)	∆H_m_ (J/g)	T_0c_ (°C)	T_c_ (°C)	∆H_c_ (J/g)	
Control	49.60	62.00	09.94	62.40	60.70	10.46	1.30
SA1	48.60	62.10	09.40	62.00	60.30	10.30	1.80
SA3	47.20	61.50	10.42	62.60	61.00	11.24	0.50
SA5	47.40	62.00	09.81	62.40	60.70	11.30	1.30
SA10	46.10	61.30	10.17	62.50	60.70	10.45	0.60
SP1	47.50	61.70	09.91	62.30	60.20	10.86	1.50
SP3	46.70	62.10	09.48	62.00	60.40	09.89	1.70
SP5	47.30	61.80	09.65	62.10	60.40	10.18	1.40
SP10	47.70	61.60	09.95	60.40	60.40	10.77	1.20

T_0m_: onset temperature of melting; T_m_: melting temperature; ∆H_m_: melting enthalpy; T_0c_: onset temperature of crystallization; T_c_: crystallization temperature; ∆H_c_: crystallization enthalpy.

**Table 4:** Drug-release and PS model parameters.

Samples	CPRD at 180 min	Parameters of PS Model				
		K_d_	K_r_	K_d_/K_r_	m	R^2^
Control	65.95 ± 4.68 ^a^	3.00 ± 0.01 ^bc^	0.67 ± 0.17 ^a^	4.67 ± 1.37 ^bd^	0.34 ± 0.01 ^de^	0.99
SA1	73.58 ± 3.91 ^b^	4.21 ± 0.21 ^abd^	0.63 ± 0.18 ^ab^	6.71 ± 2.04 ^cd^	0.37± 0.02 ^ce^	0.99
SA3	85.44 ± 2.49 ^a^	4.06 ± 0.06 ^a^	0.39 ± 0.05 ^a^	11.26 ± 1.82 ^ac^	0.39 ± 0.01 ^bc^	0.99
SA5	70.03 ± 3.01 ^b^	4.02 ± 0.20 ^a^	0.59 ± 0.12 ^a^	6.98 ± 1.09 ^c^	0.50 ± 0.01 ^a^	0.99
SA10	49.00 ± 2.85 ^c^	1.02 ± 0.02 ^d^	0.19 ± 0.05 ^b^	3.00 ± 0.51 ^cd^	0.43 ± 0.02 ^b^	0.99
SP1	72.53 ± 1.17 ^b^	4.17 ± 0.15 ^a^	0.36 ± 0.06 ^ac^	15.48 ± 2.92 ^a^	0.40 ± 0.01 ^c^	0.99
SP3	91.68 ± 3.26 ^a^	4.57 ± 0.07 ^abd^	0.35 ± 0.06 ^ac^	10.83 ± 0.89 ^abc^	0.42 + 0.16 ^abd^	0.99
SP5	50.31 ± 71.5 ^c^	3.57 ± 0.58 ^ac^	0.40 ± 0.07 ^ac^	9.13 ± 2.77 ^abc^	0.34 ± 0.02 ^de^	0.99
SP10	40.09 ± 2.29 ^d^	2.59 ± 0.57 ^bc^	0.24 ± 0.01 ^bc^	9.01 ± 2.19 ^bc^	0.39 ± 0.09 ^abcd^	0.99

Superscripts with different alphabets in the same column represent statistically significant (*p* ≤ 0.05) values.

## Data Availability

Data are available from D.B. upon request.

## Supplementary Materials

The following supporting information can be downloaded at: https://www.mdpi.com/article/10.3390/gels8080520/s1, Figure S1: Inverted tube method to confirm oleogel formation; Figure S2: Surface topology images of oleogels through the metallurgical microscope; Figure S3: Bright-field micrograph of oleogels displaying fiber morphology of fat crystals; Figure S4: XRD of (a) SA-incorporated oleogels and (b) SP-incorporated oleogels, and (c) quantification of % crystallinity among the oleogels; Figure S5: FTIR spectra of raw components used for oleogel synthesis; Figure S6: FTIR spectra of (a) SA-incorporated oleogels and (b) SP-incorporated oleogels; Figure S7: Crystallization curves of (a) SA-incorporated oleogels and (b) SP-incorporated oleogels; Figure S8:DSC thermograms of emulsifier-incorporated oleogels; Figure S9: Stress-relaxation profile of (a) SA-incorporated oleogels and (b) SP-incorporated oleogels; Table S1: Parameters of stress-relaxation curve.
