# Supplementary material for: Effects of Sorbitan Monostearate and Stearyl Alcohol on the Physicochemical Parameters of Sunflower-Wax-Based Oleogels
Source: Gels. 2022 Aug 19;8(8):520. doi: 10.3390/gels8080520 (PMC9407308; doi:10.3390/gels8080520)
Supplement: Supplementary file 1 [file gels-08-00520-s001.zip › gels-1828156-supplementary.pdf]

gels

Effects of Sorbitan Monostearate and Stearyl Alcohol on the Physicochemical Parameters of Sunflower-Wax-Based Oleogels

1. Preparation of oleogels

All the oleogel formulation appeared whitish with a good spreadability when touched with hands. The molten oleogel samples were kept at 25 °C, post which they were evaluated for their successful oleogel formation. This was done through the tube inversion method (Figure S1), where the formulated gels were kept inverted in glass bottles. Once the oleogel were formed, they were stored at 4 °C.

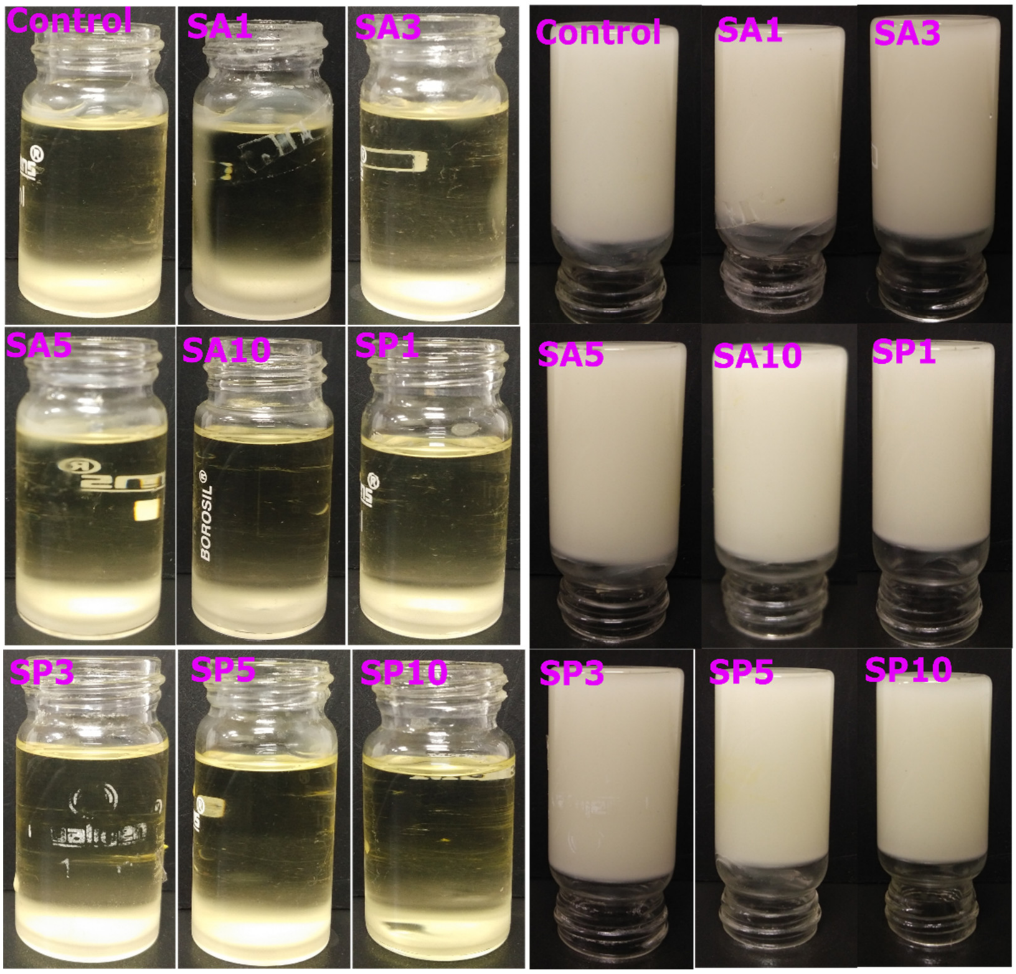

Figure S1. Inverted tube method to confirm oleogel formation.

2. Microscopy

2.1 Surface Topology

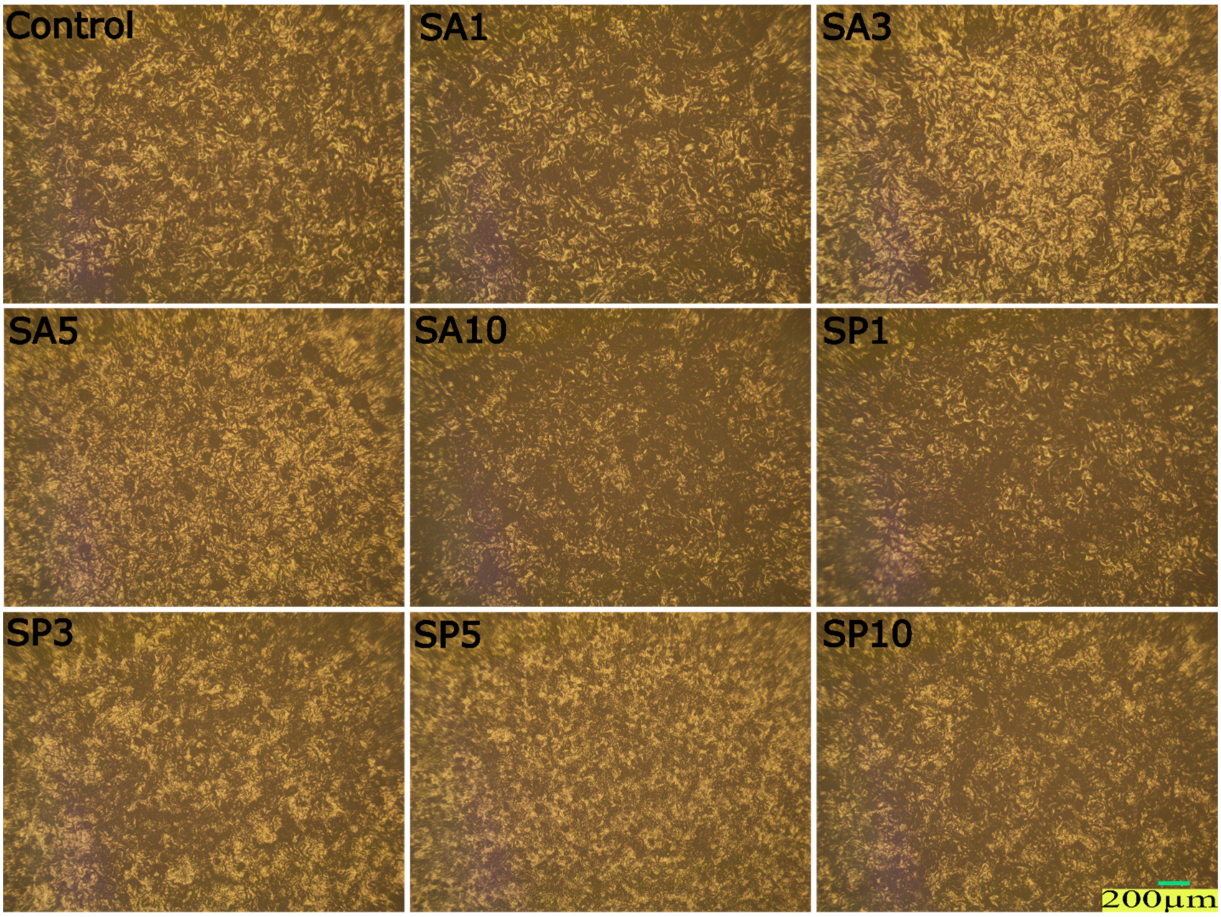

Figure S2. Surface topology images of oleogels through the metallurgical microscope.

2.2 Microstructural arrangement

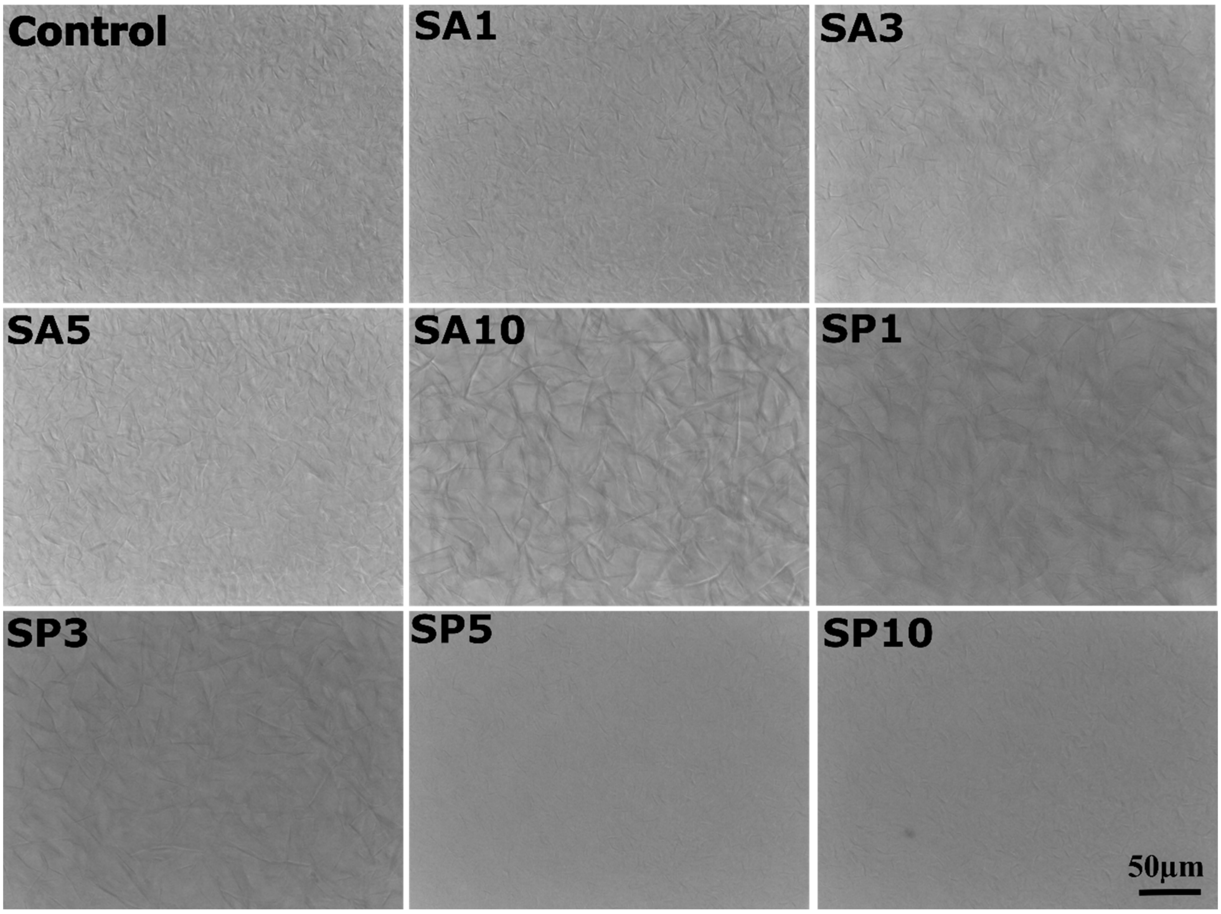

Figure S3. Bright-field micrograph of oleogels displaying fiber morphology of fat crystals.

3. XRD analysis

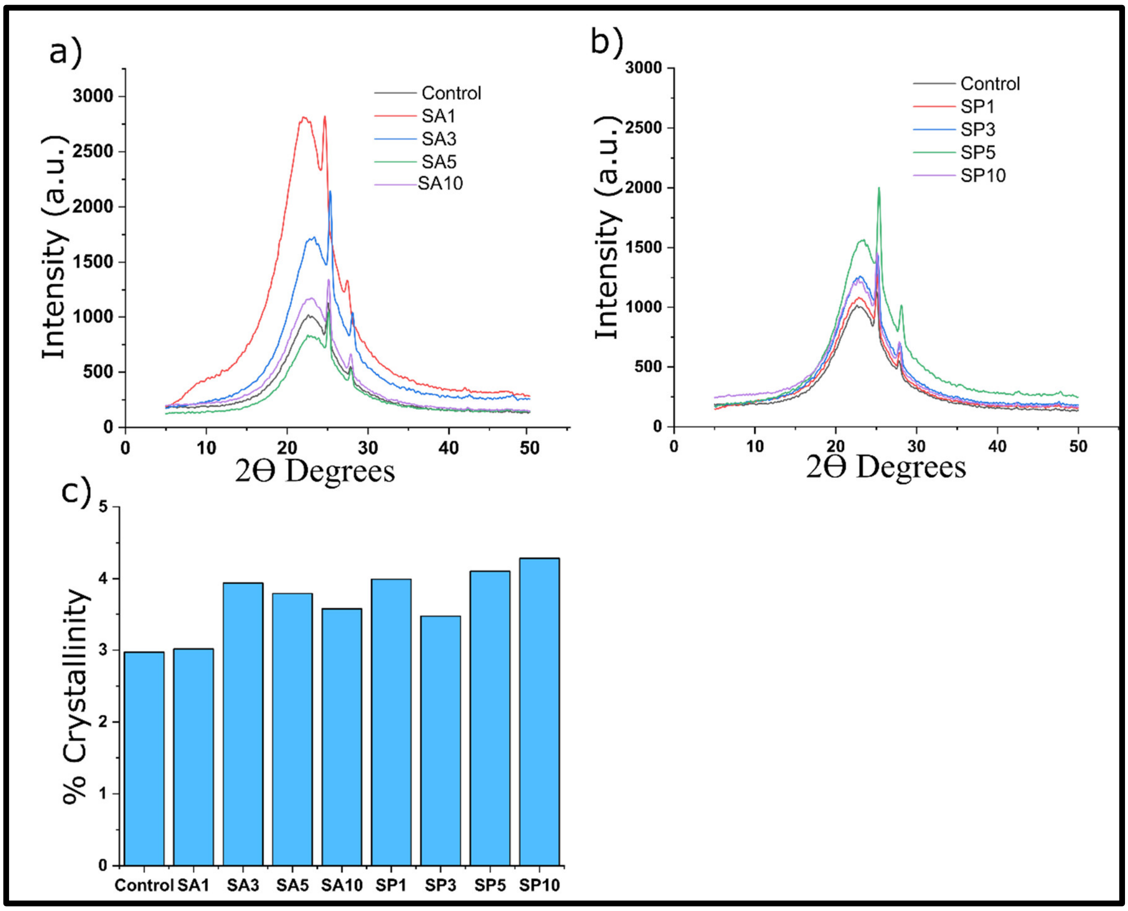

Figure S4. XRD of a) SA b) SP incorporated oleogels; c) Quantification of % crystallinity among the oleogels.

4. FTIR Analysis

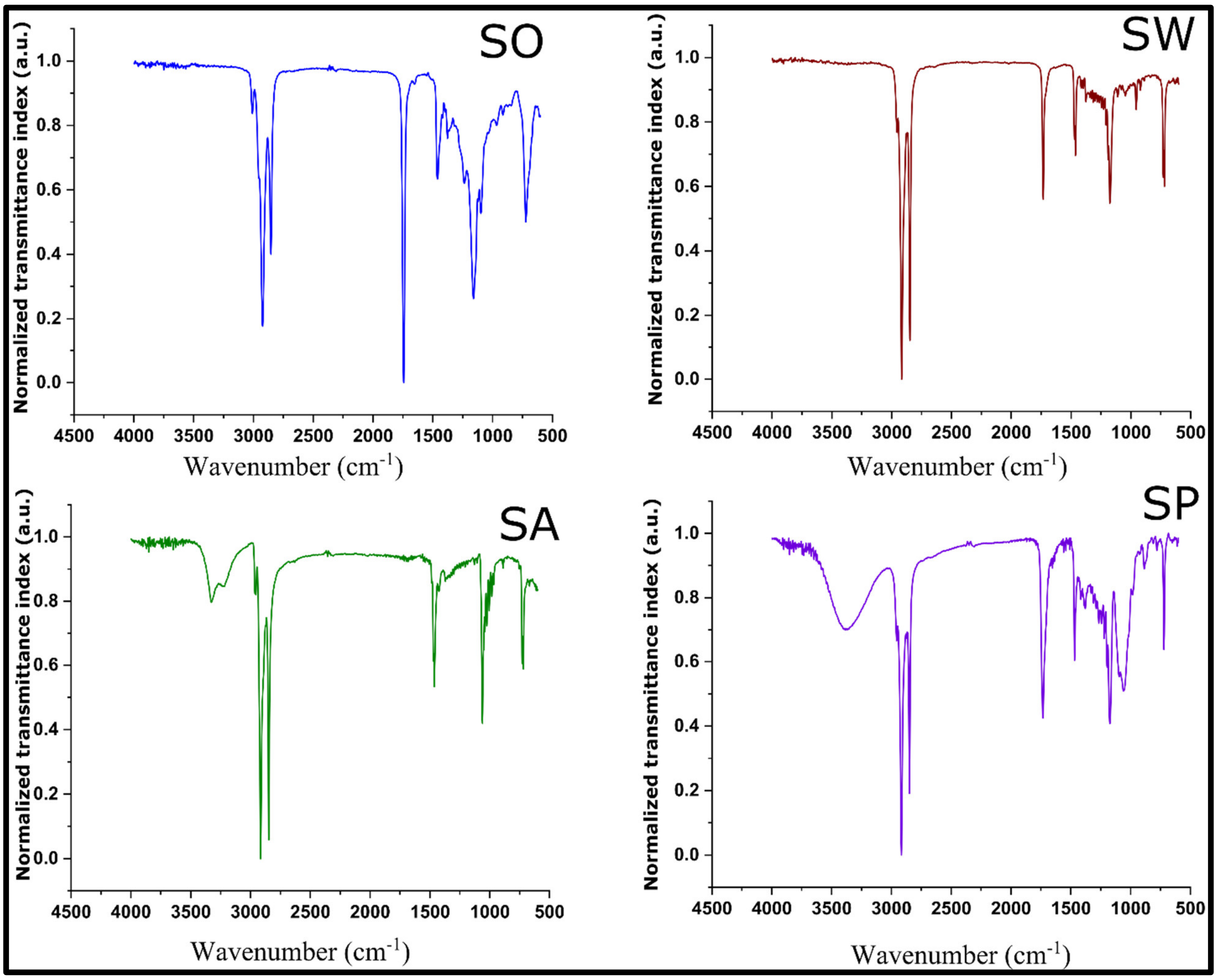

Figure S5. FTIR spectra of raw components used for oleogel synthesis.

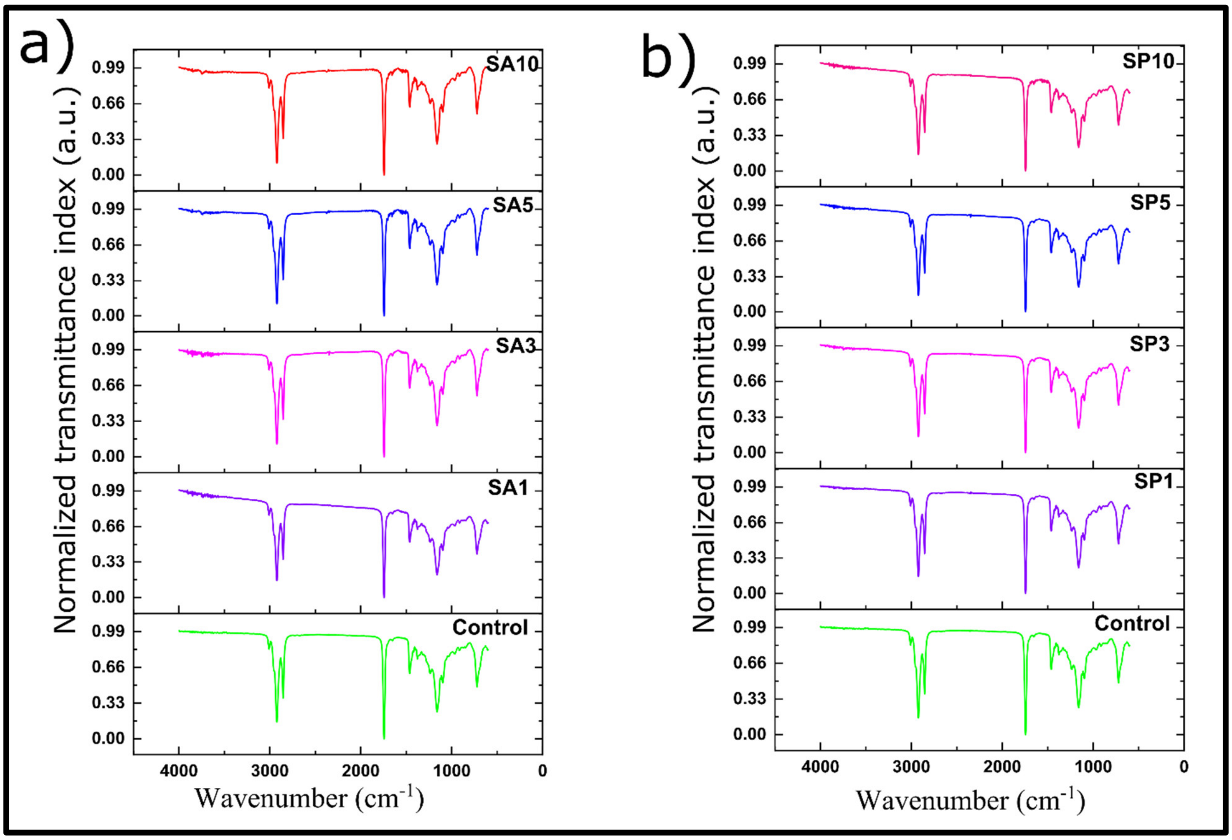

Figure S6. FTIR spectra of a) SA incorporated oleogels, and b) SP incorporated oleogels.

5. Crystallisation kinetics

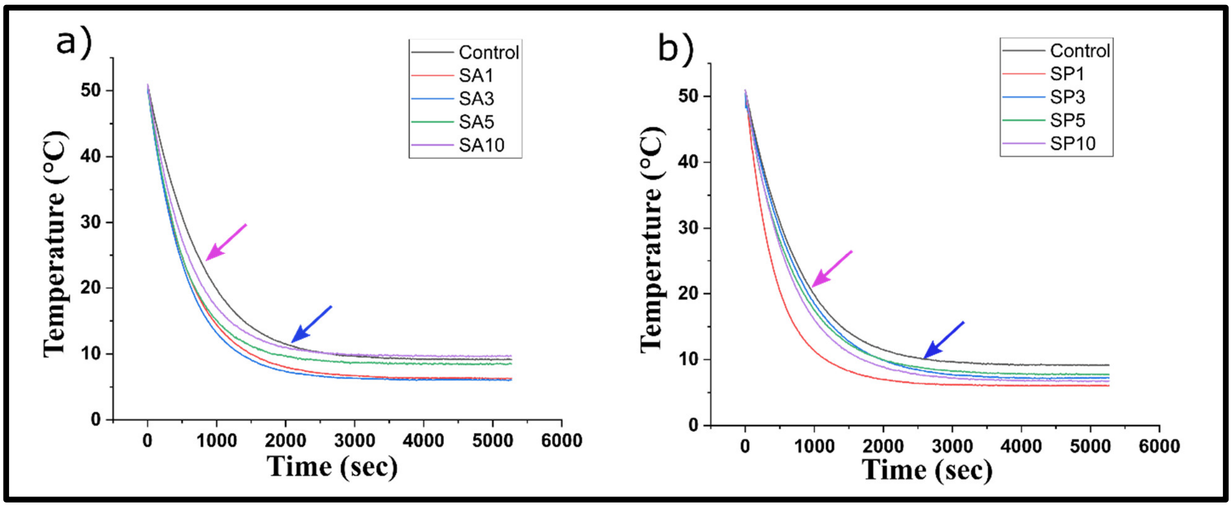

Figure S7. Crystallisation curves a) SA incorporated oleogels b) SP incorporated oleogels.

6. Differential Scanning Calorimetry

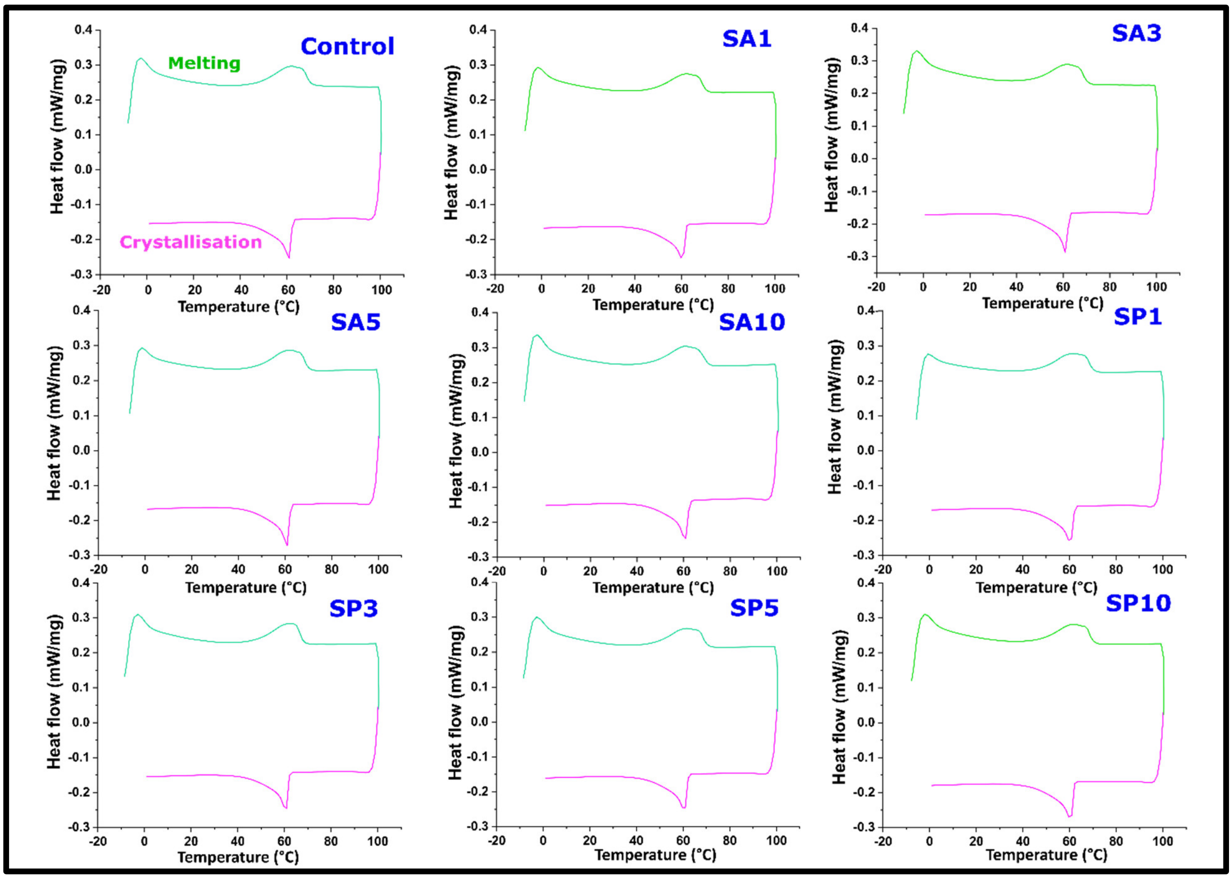

Figure S8. DSC thermograms of emulsifier incorporated oleogels.

7. Mechanical Analysis

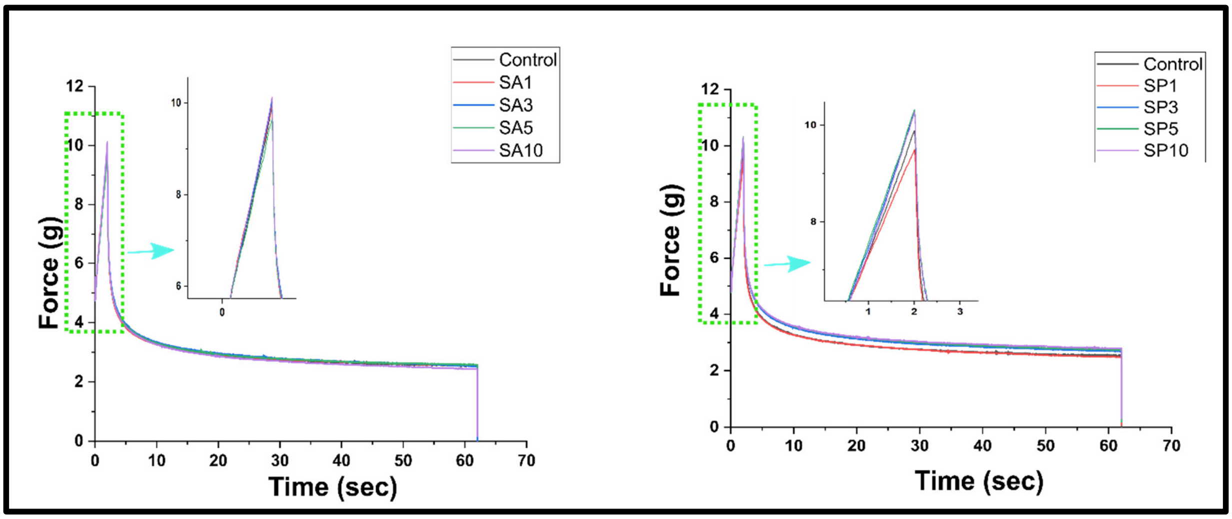

Figure S9. Stress relaxation profile of a) SA incorporated oleogels b) SP incorporated oleogels.

Supplementary Table S1. Parameters of stress relaxation curve.

Formulation	F_0	F_R	%SR
**Control**	9.98 ± 0.76	2.46 ± 0.27	75.27 2.12
**SA1**	10.21 ± 0.64	2.66 ± 0.24	74.00 1.56
**SA3**	10.16 ± 0.61	2.55 ± 0.23	74.86 2.56
**SA5**	10.04 ± 0.77	2.49 ± 0.25	75.59 3.29
**SA10**	10.18 ± 0.51	2.48 ± 0.29	73.67 2.73
**SP1**	9.49 ± 0.54	2.49 ± 0.24	72.67 2.73
**SP3**	10.07 ± 0.55	2.76 ± 0.19	72.55 2.33
**SP5**	10.08 ± 0.46	2.70 ± 0.15	73.24 1.44
**SP10**	10.57 ±0.92	2.72 ± 0.22	74.07 3.21
